# Ten simple rules for leading a successful undergraduate-intensive research lab

**DOI:** 10.1371/journal.pcbi.1011994

**Published:** 2024-04-11

**Authors:** KJE Hickman, Geoffrey Zahn

**Affiliations:** 1 MIT-WHOI Joint Program in Oceanography/Applied Ocean Science & Engineering, Cambridge and Woods Hole, Massachusetts, United States of America; 2 Biology Department, Utah Valley University, Orem, Utah, United States of America; Carnegie Mellon University, UNITED STATES

## Abstract

Participating in mentored research is an enormous benefit to undergraduate students. These immersive experiences can dramatically improve retention and completion rates, especially for students from traditionally underserved populations in STEM disciplines. Scientists typically do not receive any formal training in management or group dynamics before taking on the role of a lab head. Thus, peer forums and shared wisdom are crucial for developing the vision and skills involved with mentorship and leading a successful research lab. Faculty at any institution can help improve student outcomes and the success of their labs by thoughtfully including undergraduates in their research programs. Moreover, faculty at primarily undergraduate institutions have special challenges that are not often acknowledged or addressed in public discussions about best practices for running a lab. Here, we present 10 simple rules for fostering a successful undergraduate research lab. While much of the advice herein is applicable to mentoring undergraduates in any setting, it is especially tailored to the special circumstances found at primarily undergraduate institutions.

This is a *PLOS Computational Biology* Benchmarking paper.

## Introduction

Undergraduate research (UR) is a high-impact practice that has been demonstrated to benefit student learning, persistence, and career preparation [1,2]. Undergraduate research serves as a robust intervention for students from underrepresented groups who are at risk of dropping out of college [3,4]. By engaging students during their early years of study, they develop a sense of community and gain access to faculty mentors. A preliminary introduction to the research environment gives students time to develop their science identity and makes them more resilient to difficulties encountered during their educational careers [5]. The literature on positive outcomes associated with participation in UR is broad [6], encompassing large public research institutions, private institutions, and liberal arts colleges.

Faculty at Primarily Undergraduate Institutions (PUIs) face a unique set of challenges to maintain scholarly productivity and “successful” research programs. They often have fewer external funding opportunities [7] and far higher teaching loads than faculty at research-intensive (R1) universities. Many R1 institutions provide research opportunities for undergraduates by incorporating them into ongoing projects led by graduate students and/or postdocs, via short-term programs or with course-based undergraduate research experiences (CUREs, see Rule 10), which can be successful at any type of institution. However, the luxury of graduate student and postdoc labor is not available to most faculty at a PUI—instead, they must rely on the involvement of undergraduate researchers.

Working with undergraduate students themselves presents some unique challenges. Typically, graduate students have a more refined set of skills and direction when they begin mentored research. They also have more financial support and time dedicated to research. Conversely, undergraduate students generally require a high investment toward training before they can be independent researchers. This is because undergraduates are enrolled in full-time coursework and are only with the lab for a short time before they graduate and move on to careers or graduate programs.

While there is considerable overlap in practices that lead to successful labs in both R1 and PUI settings, the unique challenges of running a lab at a PUI require specialized approaches for recruiting lab members and fostering lab success. There has been rich discourse on methods to increase the health and productivity of research labs [8–11]. However, we note that much of the advice (even when about undergraduate students) has been geared toward R1 labs with postdocs, graduate students, and reduced teaching expectations for faculty. Here, we discuss some “rules” tailored to the specific challenges facing the principal investigators of research labs at PUIs, particularly at public universities that serve a diverse student body.

## Rule 1: Determine what “success” means in your lab

**Fig 1:**
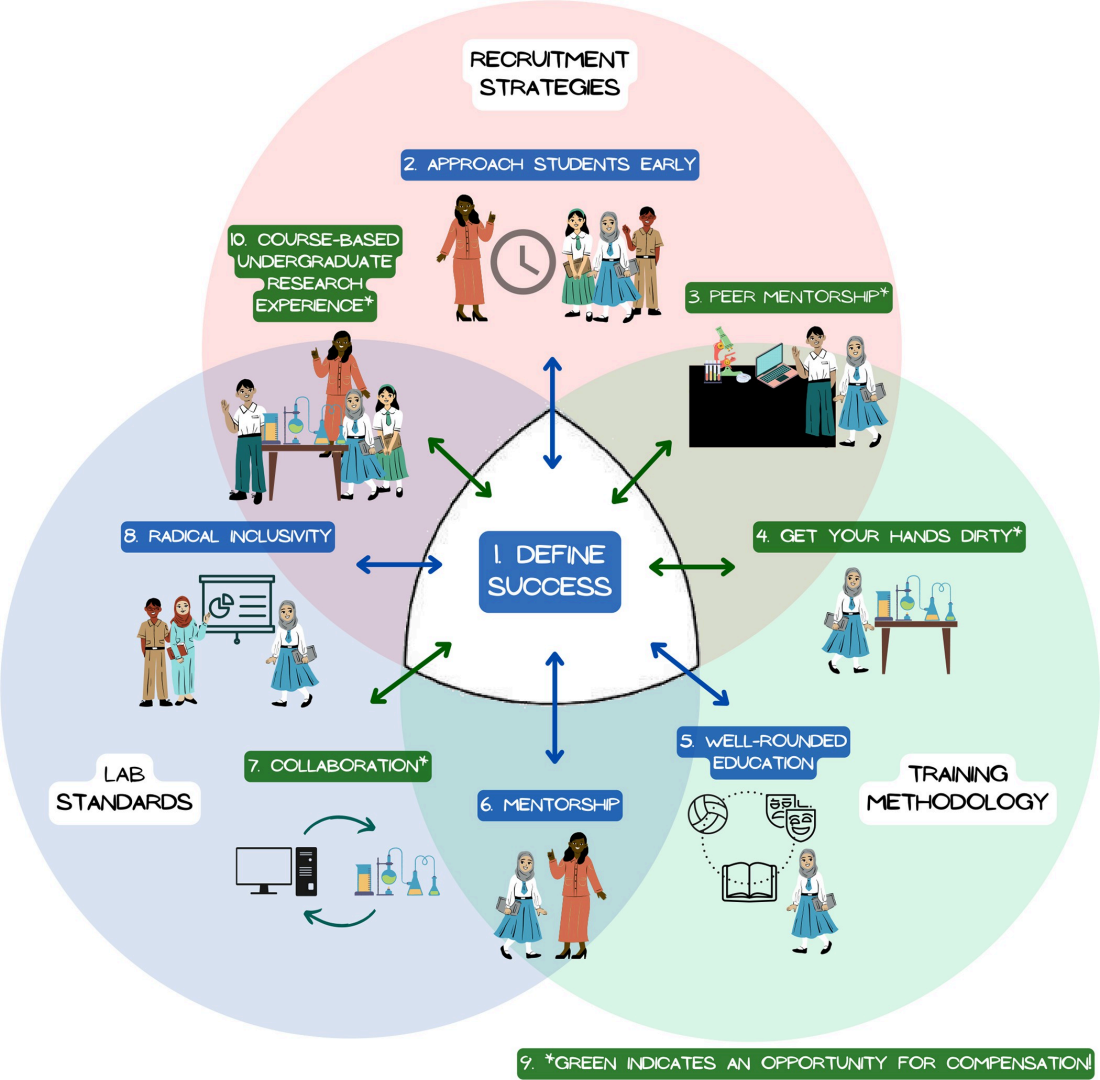
Rules that lead to lab success. Defining lab success as student success is foundational to the 9 other rules for running a successful undergraduate-intensive research lab. This definition is informed by lab standards, training methodologies, and recruitment strategies. Cultivating the principles endemic to each rule promotes student success which, in turn, provides further opportunities to strengthen the lab’s success. These mutually reinforcing processes build lab community and facilitate successful undergraduate research labs.

The crucial first step is to decide what “success” means for your PUI research lab. Setting this down in writing and communicating it to lab members will help to set the tone and focus of the lab. While external funding and publication quantity/quality are important metrics for lab “success” in some settings, we would argue that lab success at a PUI is most usefully defined as student success (Fig 1).

Student success can be measured in many ways, from retention and graduation in STEM, to increased science identity and critical thinking skills, to poster presentations, internal grant awards, and placement in graduate/professional programs. Selecting and tracking these metrics of importance will help you define your lab’s role in student success and prioritize your lab’s activities and engagement. Students who join your lab will have diverse educational and career goals, so it is imperative to have a plan in place for incorporating them into your lab’s pursuits. For example, a student planning on medical school might want to attend a different conference than one planning on graduate school, or a student considering other callings (communication, law, science policy, etc.) might benefit from altogether different career-development experiences. At all points, maintain an open dialogue with lab members about how their activities will lead to their own success in the context of the lab, and solicit feedback from each of them (individually) about what they view as “success” on short-term (approximately 3 to 6 months), medium-term (approximately 1 to 2 years), and long-term (>3 to 5 years) timescales.

The diversity of skill levels and interests you encounter with undergraduate lab members may shape your lab’s research goals in ways you did not anticipate. Some students may want to simply assist on someone else’s project, while others will be eager to start their own line of original research. Keeping a flexible research agenda to accommodate student interests and skills is fine, but undergraduates may want to push the boundaries of your lab’s unique focus beyond what you are capable of effectively supporting. Having a clear statement of “lab success” and putting lab goals in writing in a formal document will help you to guide students toward activities that support both them and your lab. An example undergraduate lab handbook has been archived online via Zenodo [12].

## Rule 2: Approach students early

Science is a multifaceted and often slow process. With the high training investment and heavy course loads characteristic of undergraduate students, the research process is slowed even further. Be prepared for things to take much longer than you expect. Actively recruiting early-stage students, even local high-school students, is a winning strategy to overcome this challenge. This will provide you with ample time to test mentoring strategies, train the students in relevant methodologies, and benefit from their application of this training. Moreover, allotting sufficient time for the students’ training will facilitate their development into independent scientists with the ability to generate and investigate their own questions and ideas.

Freshmen and sophomores in your courses may not be aware that undergraduate research is even an option. PIs at teaching-focused institutions usually have consistent access to early students through the courses they teach. Spend a bit of time in class discussing research opportunities and benefits at your institution, and use examples of student research to highlight course content. Invite your current research students to present their projects in class to help you recruit, and invite interested students to shadow in the lab for a day. Highlighting the availability and inclusivity of undergraduate research and its importance to student success can help raise awareness in student populations who otherwise may have never been told that they could be a scientist.

## Rule 3: Structure projects for peer collaboration

As a faculty member at a PUI, teaching is typically your first priority. With such restrictions on research time, a peer-mentor model can be a useful asset in your lab. This is analogous to the peer-mentor models employed by PIs at R1 institutions, with postdocs helping mentor PhD students [13]. In an undergraduate-only setting, the time and effort spent training students to serve as peer-mentors is significantly greater. After they are trained, peer-mentoring roles can be negotiated that lead to beneficial experiences for both mentor and mentee students [14]. This model can be an especially empowering role for the student mentor, developing their self-perception as a scientist. Moreover, this model develops teamwork skills and adds an element of peer-accountability which has been shown to improve retention and enjoyment of the scientific process [15].

In a PUI research setting, most students generally benefit from rotating through projects and/or duties. This variety exposes them to ideas and processes that may eventually shape their career path. Incorporating new students into senior students’ preexisting projects facilitates a flexible lab environment, which cultivates skill exploration, preparing them for independent research [16,17]. Good communication between the PI and the student research teams is also important for clearly defining roles, authorship credit, and project development. A collaborative lab environment will always be more successful than a competitive one, and you should take care to model and reinforce good collaborative practices.

## Rule 4: Get students’ hands dirty

Undergraduate students typically seek out research labs because they have a vision of what research looks like and a perception of themselves as part of this process. For example, students may visualize researchers in a white coat at the lab bench, knee-deep in a bog, or logging onto a supercomputer. There are many ways to conduct research and these variations may not be equally recognized among undergraduates. Consequently, students should be engaged throughout various steps in the research process in order to enrich their contextual understanding and experience. There is no substitute for hands-on experience. Engaging students in active research protocols early on increases retention and improves chances of attaining high-skill positions in STEM [18].

A few roles on research projects in which new students can easily participate range, for example, from data collection and entry, to student–student peer review, to computational analyses, depending on student background. As students progress, this list can expand to include more intensive responsibilities. Allowing students to participate in a broad range of scientific tasks will equip them with an applied understanding of the hidden processes in science and build early intuition for this work [19].

## Rule 5: Encourage a well-rounded education

Science is a highly creative pursuit and meaningful STEM careers can follow myriad paths. For example, a student may take interest in science communication, policy, or advocacy. Encourage your students’ diverse interests and allow them to follow their passion. This applies to the lab, their research questions, and their academic and personal life. They may want to take a ceramics class, learn to scuba dive, or spend time volunteering with campus organizations. Extracurricular activities and experiences build well-rounded individuals and more creative scientists, as well as making them more competitive applicants for jobs and postsecondary educational programs [20].

Talking to your students about their non-research passions may inspire new research paradigms or even inform how you communicate science from your lab. Promoting a healthy work/life balance and embracing the diversity of personal interests in your lab will make you more approachable and help foster an environment where lab members feel respected and fulfilled. Happy students do better science and have a positive effect on lab success.

## Rule 6: Tailor your lab to your mentorship style

Different personalities and skill sets lead to different mentorship styles. When organizing your lab, it is helpful to do some self-reflection about what sort of mentor you want to be. Developing a formal mentoring philosophy can be facilitated through mentorship training from your institution, professional societies, and government agencies. These are excellent methods to spark introspection and define your strengths, weaknesses, and goals as a mentor.

How many students can you effectively supervise? How many different ongoing projects are feasible? The right answers to these and other questions will vary for every PI. Some may be comfortable establishing a large research group with formalized peer-mentoring and defined projects. Others may do better with a small group and closer interactions with each student. It takes time to develop trust and rapport with students, and without it, they may not feel comfortable failing or asking for help. It is important to be intentional and aware of your limitations. It is also important that each student in your lab gets the individual attention that they need.

## Rule 7: Collaborate early and often

Science is inherently collaborative, and collaboration is a skill [21]. This is particularly important when running an undergraduate research lab where student training and graduation timelines do not leave much room for extensive data collection. Multiyear projects that students contribute to during their short tenure can leave most participants without tangible products to show as they apply for the next steps in their career pathway. To get things done on an undergraduate timeline, collaborations with external partners can be key.

Your lab will likely have some methodological focus that could be invaluable to other research teams. For example, if your undergraduate lab focuses on computational training, you will probably have external research labs eager for you to analyze data. Colleagues at R1 institutions often see PUI partners as a benefit for funding opportunities as well (e.g., NSF Broader Impacts). Use your professional network to advertise what your students can do and actively seek out collaborative opportunities with your academic, industry, and governmental contacts. This creates opportunities for your students to participate in projects they could not do alone, builds their professional networks, and teaches them how to be good collaborators.

## Rule 8: Practice radical inclusivity

Building an inclusive lab takes effort and commitment. Most of the excellent advice for establishing an inclusive and antiracist lab [8,11,22] is directly applicable to undergraduate research settings as well, so we will not repeat it here. However, special considerations should be noted for PUIs. For example, you will likely encounter a greater proportion of first-generation/low-income and underrepresented students at a public PUI, as each stage of the educational pipeline successively excludes more students from those populations.

Students who are the first generation in their family to attend college, who come from low-income backgrounds, and/or who identify with underrepresented groups in STEM are far less likely to approach and interact with faculty either formally or informally [23]. This makes it crucial for faculty to proactively initiate discussions and actively recruit undergraduate lab members rather than wait for students to approach them. Underrepresented students benefit more from faculty-mentored research than any other group [24] and inclusion in undergraduate research has been shown to improve these students’ persistence in STEM [25]. Find the time to meet with students from these groups, whether in your classroom or by attending extracurricular events geared toward these student groups. Invest in creating a lab environment that will support a diverse group of students and then actively recruit them early in their educational journey.

## Rule 9: Compensate students for their contributions

One of the most impactful differences between graduate and undergraduate researchers is that the latter are primarily full-time students, typically with no expectations or compensation for research activities. Finding ways to compensate undergraduates for research equalizes who can afford to participate. Your university may have internal grant mechanisms that pay or subsidize wages for undergraduate student research labor. Be proactive in finding these and other funding sources and, if paying your students is not an option, you may be able to compensate with course credit. Aside from equalizing access, compensating your students fosters mutual respect for their work/life balance which sets a precedent for students to respect their own time, manage expectations, and not overcommit.

## Rule 10: Incorporate research into your teaching

While one-on-one mentoring has the highest impact on students [19], the time investment required for this practice is not always scalable. Course-based undergraduate research experiences (CUREs) offer a way to reach more students [6]. CUREs can make research participation more inclusive and available to students who may not be aware that mentored research is an option, and they reach a “captive audience” of students who may never have considered engaging in research. It also allows a wide range of students to add meaningful research experience to their professional portfolio while earning credits toward their degree. Teaching a CURE is separate from running a research lab, but it invariably extends and informs your mentoring. The pedagogical literature has many good examples of how to design and effectively manage a CURE in your classroom [26–30].

## Conclusions

While the habits and attitudes that lead to successful research labs overlap considerably between an R1 and a PUI, there are unique features and special challenges in an undergraduate-only lab group that deserve special consideration. Here, we have tried to highlight some of the important practices that can transform those challenges into opportunities. Faculty at public PUIs play a critical role in preparing underserved students for careers in science, and often influence the types of scientists that these students will become. By teaching them how to be good scientists and collaborative community members, and how to cultivate a deep well of patience and compassion, you’re enabling their success. Framing “lab success” in terms of “student success” as a guiding principle will lead to positive outcomes for students and your lab.
